# Aristolochic acid I exposure decreases oocyte quality

**DOI:** 10.3389/fcell.2022.838992

**Published:** 2022-08-11

**Authors:** Weidong Li, Jiaming Zhang, Xiaoxia Yu, Fei Meng, Ju Huang, Liangran Zhang, Shunxin Wang

**Affiliations:** ^1^ Center for Reproductive Medicine, Cheeloo College of Medicine, Shandong University, Jinan, China; ^2^ Advanced Medical Research Institute, Shandong University, Jinan, Shandong, China; ^3^ Shandong Provincial Key Laboratory of Animal Resistance Biology, College of Life Sciences, Shandong Normal University, Jinan, Shandong, China; ^4^ National Research Center for Assisted Reproductive Technology and Reproductive Genetics, Shandong University, Jinan, Shandong, China; ^5^ Key Laboratory of Reproductive Endocrinology of Ministry of Education, Jinan, Shandong, China; ^6^ Shandong Provincial Clinical Research Center for Reproductive Health, Jinan, Shandong, China

**Keywords:** aneuploidy, oocyte, spindle assembly, mitochondrial dysfunction, aristolochic acids

## Abstract

Oocyte quality is a determinant of a successful pregnancy. The final step of oocyte development is oocyte maturation, which is susceptible to environmental exposures. Aristolochic acids (AAs), widely existing in *Aristolochia* and *Asarum* plants that have been used in traditional medicine, can result in a smaller ovary and fewer superovulated oocytes after *in vivo* exposure to mice. However, whether AAs affect oocyte maturation and the underlying mechanism(s) are unclear. In this study, we focused on the effect of Aristolochic acid I (AAI), a major compound of AAs, on the maturation of *in vitro* cultured mouse oocytes. We showed that AAI exposure significantly decreased oocyte quality, including elevated aneuploidy, accompanied by aberrant chiasma patterns and spindle organization, and decreased first polar body extrusion and fertilization capability. Moreover, embryo development potential was also dramatically decreased. Further analyses revealed that AAI exposure significantly decreased mitochondrial membrane potential and ATP synthesis and increased the level of reactive oxygen species (ROS), implying impaired mitochondrial function. Insufficient ATP supply can cause aberrant spindle assembly and excessive ROS can cause premature loss of sister chromatid cohesion and thus alterations in chiasma patterns. Both aberrant spindles and changed chiasma patterns can contribute to chromosome misalignment and thus aneuploidy. Therefore, AAI exposure decreases oocyte quality probably *via* impairing mitochondrial function.

## Introduction

A key determinant for a successful pregnancy and embryonic development is oocyte quality, which relies on the development of oocytes. The final step of oocyte development is oocyte maturation, which refers to when arrested oocytes resume meiosis and progress to metaphase II (MII). The process of oocyte maturation is particularly vulnerable to environmental pollutants and chemicals (Rhind et al., 2010; He et al., 2019). The impairment of oocyte maturation may produce poor quality of oocytes, which is the common cause of infertility, miscarriage, and congenital genetic disease (Nagaoka et al., 2012).

Aristolochic acids (AAs), a group of nitrophenanthrene carboxylic acids, are widely present in *Aristolochia* and *Asarum* plants (Debelle et al., 2008). AAs have been used to treat a variety of diseases, including arthritis and inflammation (reviewed in Zhang H. M. et al., 2019; Anger et al., 2020). However, AAs have been reported to cause aristolochic acid nephropathy (AAN), bladder cancer, and hepatocellular carcinoma (Arlt et al., 2002; Zhang H. M. et al., 2019). Therefore, products containing AAs have been prohibited in many countries. However, herb preparations containing AAs from *Aristolochia* and *Asarum* are still used in many regions of the world (Gold and Slone, 2003; Grollman, 2013). In addition, AAs can persistently contaminate soil and bioaccumulate in crops (Chan et al., 2016; Li et al., 2018). AAs are mainly composed of 8-methoxy-6-nitro-phenanthro-(3,4-d)-1,3-dioxolo-5-carboxylic acid (AAI) and 6-nitro-phenanthro-(3,4-d)-1,3-dioxolo-5-carboxylic acid (AAII) (Shibutani et al., 2007). Both AAI and AAII show genotoxic and carcinogenic effects by forming DNA adducts, and AAI can also cause nephrotoxicity probably *via* inducing oxidative stress and apoptosis (Jadot et al., 2017).

Meiosis is a specialized type of cell division through which germline cells produce gametes. In males, meiosis is a continuous process that occurs in waves throughout adulthood to produce round spermatids which develop into sperm. However, in females, oocytes initiate meiosis during fetal development. After completing crossover (CO) recombination, oocytes are arrested for a prolonged period at the diplotene/dictyate stage of meiotic prophase I, morphologically identified by a large nucleus which is also called a germinal vesicle (GV). From puberty, in response to luteinizing hormone (LH), a small proportion of fully grown GV oocytes resume meiosis, which is indicated by GV breakdown (GVBD). At metaphase I (MI), homologous chromosomes (homologs) are pulled by spindle microtubules to align on the equatorial plate. After CO completion and synaptonemal complex (SC) disassembly, homologs are connected by chiasmata, the cytological manifestation of COs, which can be easily visualized at MI. The first polar body extrusion (PBE) indicates the completion of meiosis I. The oocyte then proceeds to metaphase II (MII) where it is arrested. Once fertilized, the oocyte resumes meiosis and two pronuclei are formed. Thereafter, the two pronuclei fuse, and the zygote divides mitotically to form a two-cell embryo (Morelli and Cohen, 2005; Bolcun-Filas and Handel, 2018), which further develops into a blastocyst (Zhu and Zernicka-Goetz, 2020).

In spite of the warning that has been given, traditional medicines containing AAs still prevail in many countries, which increases the AAs exposure (Jadot et al., 2017). Moreover, AAs can contaminate soil, food, and water (Chan et al., 2016; Li et al., 2018; Drăghia et al., 2021). The *in vivo* exposure of AAI in mice decreases the weight of the body and ovaries by inducing apoptosis, and also decreases the number of superovulated follicles in females (Kwak et al., 2014), suggesting that AAI most likely affects oocyte development. AAI exposed to porcine oocytes seems to impair oocyte maturation and cause aberrant distribution/morphologies of the spindle and mitochondria (Zhang Y. et al., 2019). However, how AAI impairs oocyte maturation is unclear. Importantly, oocyte (and thus embryo) aneuploidy is the major cause of human miscarriage and infertility. Whether and how AAI exposure increases oocyte aneuploidy is also worth further investigation. Accordingly, this study aimed to explore the effect of AAI on oocyte maturation and aneuploidy, and the underlying mechanisms using mouse oocytes as an *in vitro* model.

## Materials and methods

### Animals

ICR mice were raised in a temperature-controlled animal room with a 12 h light/dark cycle and fed with a regular mice diet (SFS9112, Xie-tong biomedicine, China), which is formulated with ∼20% protein and ∼12% fat, and also supplemented with multiple vitamins and minerals. ICR female mice (4–6 week-old) were used in this study. The care protocols and usage of mice in this study were reviewed and approved by the Animal Ethics Committee of the School of Medicine, Shandong University.

### Oocyte collection and *in vitro* culture

Female mice were subjected to superovulation and oocytes were collected as previously described (Li et al., 2019; Li et al., 2020). Briefly, 4–6 week-old ICR female mice were intraperitoneally injected with 10 IU pregnant mare serum gonadotropin (PMSG, NSHF, China) diluted in normal saline. After 48h, ovaries were surgically collected in the M2 medium (M7167, Sigma-Aldrich, United States) supplemented with 2.5 μM milrinone (HY-14252, MCE, United States). Collected ovaries were then gently and repeatedly punctured with a 1 ml syringe needle to release the cumulus-oocyte complexes (COCs). Morphologically intact COCs with ≥3 layers of granulosa cells were picked with a pipette under a stereomicroscope (SZ61, Olympus). After removal of granulosa cells, these fully grown GV oocytes were collected by mouth pipette and washed with milrinone-free M2 medium. ∼20 GV oocytes per group were transferred to ∼30 μl pre-warmed M16 medium (M7292, Sigma-Aldrich, United States), which was covered by paraffin oil to prevent medium evaporation, and cultured at 37°C under a humidified atmosphere with 5% CO_2_. Under this standard culture condition, oocytes develop to GVBD, metaphase I (MI), and metaphase II (MII) stages after 2, 8, and 12 h, respectively (Li S. et al., 2012). Samples were collected at appropriate time points to evaluate oocyte quality.

Oocytes used in this study: 105 MII oocytes (6 mice) for *in vitro* fertilization and two-cell embryo examination; 456 MII oocytes (18 mice) for *in vitro* fertilization and blastocyst examination; 278 GV oocytes (12 mice) and 395 oocytes (20 mice) for GVBD and PBE detection, respectively; 165 MII oocytes (12 mice) for chromosome spread to evaluate aneuploidy and 110 MI oocytes (12 mice) to count chiasmata; 182 MI oocytes (9 mice) for spindle assembly and chromosome alignment experiments; 300 GV oocytes (12 mice), 300 GVBD oocytes (12 mice), 300 MI oocytes (12 mice), and 300 MII oocytes (12 mice) for RT-qPCR to detect gene expression in different stages, respectively; 187 oocytes (9 mice) and 184 oocytes (9 mice) for GVBD and MI mitochondrial membrane potential assay, respectively; 120 oocytes (6 mice) for GVBD and MI ATP level measurement, respectively; 343 oocytes (12 mice), 322 oocytes (12 mice), and 313 oocytes (12 mice) for ROS level measurement in GVBD, MI, and MII stages, respectively; 324 oocytes (12 mice), 326 oocytes (12 mice), and 385 oocytes (12 mice) for early apoptosis detection in GVBD, MI, and MII stages, respectively; 60 GVBD oocytes (6 mice) and 40 MI oocytes (6 mice) for RNA-seq.

### Aristolochic acid I treatment

Aristolochic acid I (AAI) (A5512, Sigma-Aldrich, United States) was dissolved in DMSO (D2650, Sigma-Aldrich, United States) to make a 100 mM stock solution and then diluted in the M16 medium to make a working solution. According to previous publications, a cumulative dose of ∼350–400 mg of AAs can trigger Balkan endemic nephropathy (BEN) (Li et al., 2018) and cumulative ingestion of ≥250 mg of AAs increases the risk of urothelial carcinomas of the upper urinary tract in Taiwanese patients (Hoang et al., 2016). If these AAs enter the blood, the concentration of AAs in the blood is 200–300 μM. Based on this estimation, 0 μM, 25 μM, 50 μM, and 100 μM AAI were used in this study.

### Immunofluorescence and confocal microscopy

Oocytes were fixed in 4% formaldehyde solution (P0099, Beyotime, China) at room temperature for 30 min. After being washed 3 times using 0.1% polyvinyl alcohol (PVA)-PBS, the oocytes were permeabilized in 0.5% Triton X-100 diluted with Dulbecco’s Phosphate Buffered Saline (D-PBS) at room temperature for 20 min. For immunostaining, samples were first blocked with 1% bovine serum albumin (BSA, A1933, Sigma-Aldrich, United States) at room temperature for 1h, and then incubated at 4°C for 2 h with a FITC-conjugated anti-α-tubulin mouse monoclonal antibody (1:500, F2168, Sigma-Aldrich, United States), which specifically recognizes α-tubulin (Zhu et al., 2018; Han et al., 2020). After being washed three times with D-PBS at room temperature in the dark, nuclei were stained with DAPI (10ug/ml, E607303, Sangon, China) at room temperature for 10 min. Oocytes were mounted on glass slides with antifade solution (S2100, Solarbio, China). For each oocyte to be imaged, the top and the bottom were determined and 7 evenly spaced z-sections were captured using z-stacks under a rotary laser confocal microscope (Dragonfly, Andor Technology) driven by Fusion Software. Images were presented as maximum intensity projections of the middle 5 frames (excludes the first one and the last one since these two sections have little information).

### Quantitative reverse transcription PCR (RT-qPCR)

For each experiment per treatment, RNA was extracted from 25 oocytes using RNeasy Mini Kit (74104, Qiagen) and reverse transcribed into cDNA using Hiscript II Q RT Supermix (R223, Vazyme, China). universal SYBR Green fast qPCR Mix (RK21203, ABclonal, China) was used for real-time fluorescence quantitative detection with Roche LightCycler 480 detection system (Roche Applied Science). PCR primers were listed in Supplementary Table S1. PCR was performed in a 10 μl reaction system (1 μl cDNA template; 5 μl SYBR Green Mix; 0.25 μl 10 μM forward primer and 0.25 μl 10 μM reverse primer; 3.5 μl ddH_2_O) using the following amplification condition: pre-denaturation at 95°C for 3 min, 45 cycles of denaturing at 95°C for 5 s and annealing at 60°C for 34 s. Each experiment was performed with three replicates and the cycle threshold (Ct) value was set between 20 and 30. *Gapdh* was used as the internal control. The gene expression level relative to *Gapdh* was calculated by the 2^−△△Ct^ method (Livak and Schmittgen, 2001).

### Chromosome spread

Oocyte chromosome spread was performed as described previously (Li et al., 2019; Li et al., 2020). Briefly, the zona pellucida of oocytes were removed with hydrochloric acid in the M2 medium (1/500 dilution). This process was monitored in real-time under the stereomicroscope (SZ61, Olympus). When the zona pellucida nearly disappeared, oocytes were washed three times with 0.1% PVA-PBS solution to prevent adhesion, transferred to an adhesive slide (188105, Citotest, China), and lysed in alkaline hypotonic solution (1% PFA, 0.15% Triton X-100, 3 mM dithiothreitol, pH = 9.2). After air dried, slides with samples were stored at -20°C or used for staining directly. For staining, slides were washed three times using PBS and stained with DAPI (10ug/ml, C1002, Beyotime, China) or PI (10ug/ml, ST512, Beyotime, China) for 20 min at room temperature in the dark. A drop of antifade (S2100, Solarbio, China) was applied, a coverslip was added, and then the slide was sealed with nail polish. Images were captured under a rotary laser confocal microscope (Dragonfly, Andor Technology).

### Mitochondrial membrane potential (MMP) assay

MMP was determined by the JC-1 assay using a commercial kit (C2006; Beyotime, China) according to the manufacturer’s instructions. JC-1 is a lipophilic cationic fluorescent dye. JC-1 enters the mitochondrial matrix and forms aggregates that emit red fluorescence (the maximal excitation wavelength Ex = 585 nm and emission wavelength Em = 590 nm), however, JC-1 monomers outside of the mitochondrial matrix emit green fluorescence (maximal Ex = 514 nm and Em = 529 nm). MMP is positively correlated with the level of JC-1 aggregates and thus can be measured by the ratio of red to green fluorescence (Sivandzade et al., 2019). In brief, 50 µl JC-1 stock solution was diluted with 8 ml ddH_2_O by vigorous vortex and then 2 ml JC-1 staining buffer was added to make the working solution. ∼15 oocytes per group were incubated with a 30 μl JC-1 working solution covered by paraffin oil at 37°C for 20 min and then washed with 0.1% PVA-PBS. All images were acquired with the same laser intensity and exposure time under an inverted fluorescence microscope (IX71, Olympus) with Olympus fluorescence mirror units U-FBWA (for JC-1 green; Ex = 469 ± 18 nm; Em = 525 ± 20 nm) or U-FGWA (for JC-1 red; Ex = 560 ± 20 nm; Em = 630 ± 35 nm). The fluorescence intensity of each cell was quantified using ImageJ (NIH). A region near the target cell was randomly picked and its fluorescence intensity was measured and considered as the background. The fluorescence intensity of a cell = (cell area x average pixel intensity of this cell)—(cell area x average background pixel intensity). MMP was evaluated as the ratio of the red to green fluorescence intensity.

### Adenosine 5′-triphosphate (ATP) content detection

ATP content was determined by the classical firefly luciferase using an Enhanced ATP Assay Kit (S0027, Beyotime, China). In the presence of ATP and Mg^2+^, firefly luciferase oxidizes luciferin to oxyluciferin which emits luminescence (Inouye, 2010). For each experiment per treatment, 10 oocytes were collected in a 0.2 ml centrifuge tube with a 4 μl lysing solution. After lysed by three rapid freeze-thaw cycles, 16 μl enzyme working solution was added and the mixture was transferred into an opaque 96-well plate, which was pretreated with enzyme working solution to eliminate possible ATP contamination. Luminescence intensity was measured using the luminometer (EnSpire, PerkinElmer, 0.01 p.m. sensitivity). ATP concentration was determined by matching the luminescence intensity to a standard curve generated from 5 different ATP concentrations (0.02, 0.04, 0.06, 0.08, and 0.1 nM) according to the manufacturer’s instruction.

### ROS detection

ROS level was determined with the reliable probe DCFH-DA (2′,7′-Dichlorodihydrofluorescein diacetate) using a ROS assay Kit (S0033S, Beyotime, China) according to the manufacturer’s instruction. Briefly, the DCFH-DA stock solution was diluted (1/1,000) with the M2 medium to make a 10 µM working solution. ∼10 oocytes per treatment in each experiment were incubated with 30 μl DCFH-DA working solution in the dark at 37°C for 20 min (Hempel et al., 1999). After being washed with 0.1% PVA-PBS, samples were transferred to a coverglass-bottom dish. Images were acquired under an inverted fluorescence microscope (IX71, Olympus). Fluorescence intensity was quantified using ImageJ as described in the MMP assay.

### Annexin V staining assay

Early apoptosis of oocytes was examined using the Annexin V-FITC Apoptosis Kit (C1062S, Beyotime, China). ∼30 oocytes were incubated with 30 μl detection solution (5 μl Annexin V-FITC diluted in 195 μl Annexin V-FITC binding buffer) in the dark at room temperature for 15 min. Oocytes were then transferred to a coverglass-bottom dish. Images were captured using a rotary laser confocal microscope (Dragonfly, Andor Technology). Oocytes with green fluorescence on the cytoplasmic membrane were considered early apoptotic oocytes (He et al., 2019).

### *In vitro* fertilization (IVF) and early embryo culture

Cumulus-oocyte complexes (COCs) with ≥3 layers of granulosa cells were isolated from 4 to 6 week-old female mice by puncturing the ovary with a syringe needle. After granulosa cells were removed, GV oocytes were washed with the M2 medium (M7167, Sigma-Aldrich, United States) and transferred into the M16 medium supplemented with 10% FBS (10100147, Gibco, United States), 50 mIU/ml FSH (5925-FS-010, R&D Systems, United States) and 1 μg/ml 17β-estradiol (E8875, Sigma-Aldrich, United States). After being cultured for 12 h at 37°C in the atmosphere with 5% CO_2_, the oocytes with the first polar body were transferred to HTF medium (MR-070, Millipore, United States) and incubated with capacitated spermatozoa at 37°C in the atmosphere with 5% CO_2_. After 6 h, fertilized oocytes (with two pronuclei) were examined. All oocytes were then transferred into a pre-balanced KSOM medium (MR-121, Millipore, United States) at 37°C in a humidified atmosphere with 5% CO_2._ After 18h, two-cell embryos were examined. After another 72 h, blastocysts were examined.

### RNA-seq analysis

For each experiment, 120 GV oocytes from 4 to 6 mice were divided into two groups, cultured in the M16 medium with or without 50 μM AAI for 2 h or 8 h to reach GVBD or MI, respectively. For each group, 5–10 oocytes were used for RNA-seq, which was carried out on a BGISEQ-500 sequencing platform by Shenzhen Huada Gene Technology Co. For each experiment, at least 3 repeats were done for the control and treatment, respectively. FeatureCounts (v2.0.1) was used to count reads of genes. DESeq2 was used to determine the differentially expressed gene by the standard threshold of “*padj* < 0.05 and |log2FoldChange| > 1” (Liao et al., 2014; Love et al., 2014; Zhang F. L. et al., 2019). ClusterProfiler (v4.0.1) and Metascape (http://metascape.org) were used to perform GO and KEGG pathway analysis (Zhou et al., 2019; Wu et al., 2021). Notably, the critical value for a valid GO term or KEGG signaling pathway was *p-value* < 0.05. Fragments Per Kilobase of exon model per Million mapped fragments (FPKM) was used to normalize the expression level of mRNA (Zhang F. L. et al., 2019). RNA-seq data are available at NCBI (SRA Bioproject, accession number PRJNA793336, and PRJNA836407).

### Statistical analysis

**FIGURE 1:**
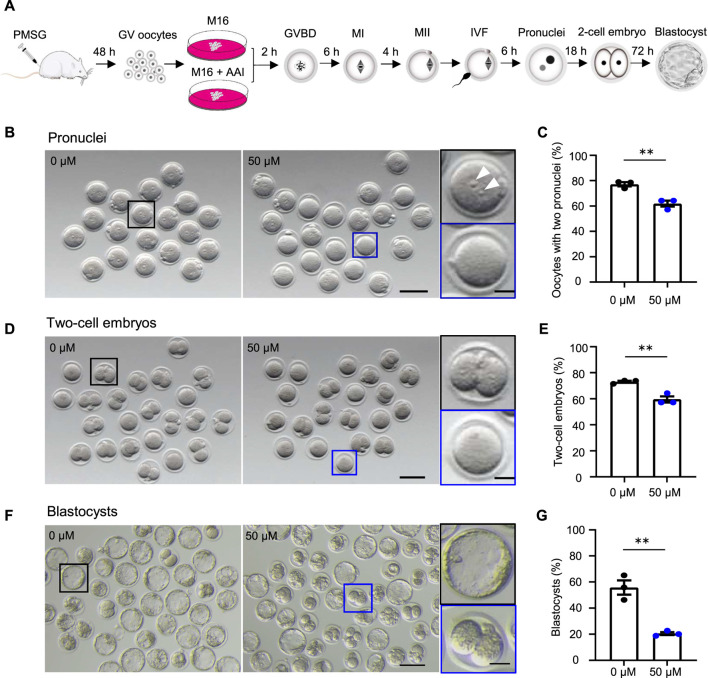
AAI exposure to oocytes decreases the fertilization rate and embryonic development potential in mice. **(A)** Schematic of experimental design. **(B–G)** The fully grown GV oocytes retrieved from mouse ovaries were matured in the medium supplemented with 0 or 50 μM AAI for 12 h to reach the metaphase II (MII) stage. The MII oocytes were performed *in vitro* fertilization (IVF) for 6 h and two pronuclei were examined **(B,C)**. All oocytes were then transferred into an embryonic culture medium and cultured for 18 h and two-cell embryos were examined **(D,E)**. After being cultured for another 72h, blastocysts were examined **(F,G)**. **(B)** Representative images showing the two pronuclei after IVF. Scale bar, 100 μm. Black box and the enlarged picture showing an MII oocyte with two pronuclei (top, white arrowheads); blue box and the enlarged picture showing an MII oocyte without a pronucleus (bottom). Scale bar in the enlarged picture, 25 μm. **(C)** The percentage of oocytes with two pronuclei in **(B)**. **(D)** Representative images showing two-cell embryos. Scale bar, 100 μm. Black box and the enlarged picture showing a two-cell embryo (top); blue box and the enlarged picture showing a zygote or an unfertilized oocyte (bottom). Scale bar in the enlarged picture, 25 μm. **(E)** The percentage of two-cell embryos in **(D)**. **(F)** Representative images showing the blastocysts. Scale bar, 100 μm. Black box and the enlarged picture showing a blastocyst (top); blue box and the enlarged picture showing a two-cell embryo (bottom). Scale bar in the enlarged picture, 25 μm. Error bar, mean ± SEM of 3 independent experiments **(C**,**E**,**G)**. Totally, 56 and 49 MII oocytes from 0 to 50 μM AAI treatments for IVF, two pronuclei, and two-cell embryos, respectively **(C,E)**. 140 and 316 MII oocytes from 0 to 50 μM AAI treatments for IVF and blastocysts, respectively **(G)**. **, *p* < 0.01; two-tailed *t*-test.

**FIGURE 2:**
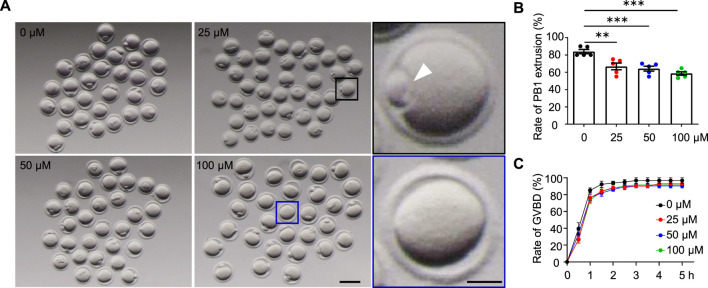
AAI exposure disrupts the first polar body (PB1) extrusion in mouse oocytes. **(A)** The GV oocytes were matured in the M16 medium with 0, 25, 50, and 100 μM AAI for 12 h to reach the MII stage. Representative images showing PB1 extrusion. Scale bar, 100 μm. Black box and the enlarged picture showing an oocyte with PB1 (PB1, white arrowhead). Blue box and the enlarged picture showing an oocyte without PB1. Scale bar in the enlarged picture, 25 μm. **(B)** The quantification of PB1 extrusion rate in **(A)**. Error bar, mean ± SEM of 5 independent experiments. ∼20 GV oocytes per treatment in each experiment and totally, 104, 105, 94, and 92 GV oocytes for 0, 25, 50, and 100 μM AAI treatments, respectively. Only comparisons with significant differences were indicated; **, *p* < 0.01; ***, *p* < 0.001; one-way ANOVA and the LSD test. **(C)** After GV oocytes were matured in the M16 medium with 0, 25, 50, and 50 μM AAI for the indicated time, GVBD rates were calculated. Error bar, mean ± SEM of 3 independent experiments. GVBD rates seemed to be slightly decreased in AAI treatment groups compared with the control but no significant difference was detected. ∼20 GV oocytes per treatment in each experiment. Totally, 70, 70, 69, and 69 GV oocytes for 0, 25, 50, and 100 μM AAI treatments, respectively.

**FIGURE 3:**
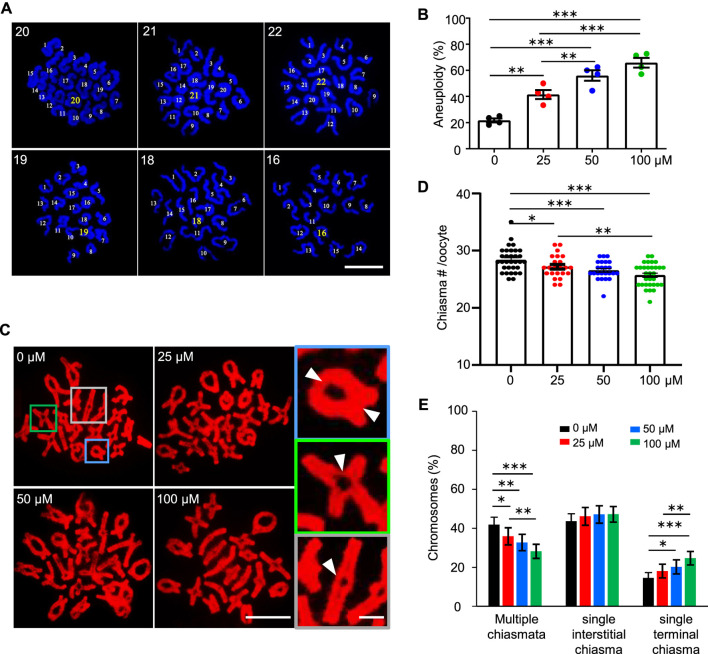
AAI exposure induces a high frequency of aneuploidy in mouse oocytes. **(A)** The GV oocytes were matured in the M16 medium with 0, 25, 50, and 100 μM AAI for 12 h to reach MII. Representative images showing MII oocyte spread chromosomes with DAPI staining. The number of chromosomes in each nucleus was indicated. Scale bar, 10 μm. **(B)** Quantification of aneuploidy frequency in **(A)**. 8–17 MII oocytes per treatment in each experiment. Totally, 51, 35, 46, and 33 MII oocytes were examined in 0, 25, 50, and 100 μM AAI treatments, respectively. Error bar, mean ± SEM of 4 independent experiments. **(C)** The GV oocytes were matured in the M16 medium with indicated AAI concentrations for 8 h to reach metaphase I (MI). Representative images showing MI oocyte spread chromosomes stained with PI. Scale bar, 10 μm. At MI, chiasmata connect the homologous chromosomes. The enlarged pictures on the right show the homologous chromosomes connected with 2 chiasmata (top), a single interstitial chiasma (middle), or a single terminal chiasma (bottom). White arrowheads indicating the positions of chiasmata. Scale bar in the enlarged picture, 5 μm. **(D)** Quantification of chiasmata numbers per nucleus in **(C)**. 32, 23, 24, and 31 MI oocytes from 0, 25, 50, and 100 μM AAI treatments, respectively. Error bar, mean ± SEM. **(E)** Quantification of the proportions of chromosomes with different classes of chiasmata in **(C)**. 640, 460, 480, and 600 MI homologous chromosomes from 0, 25, 50, and 100 μM AAI treatments, respectively. Error bar, 95% confidence interval. Only comparisons with significant differences were indicated; *, *p* < 0.05; **, *p* < 0.01; ***, *p* < 0.001 **(B,D,E)**; one-way ANOVA and the LSD test **(B,D)**; Chi-square **(E)**.

**FIGURE 4:**
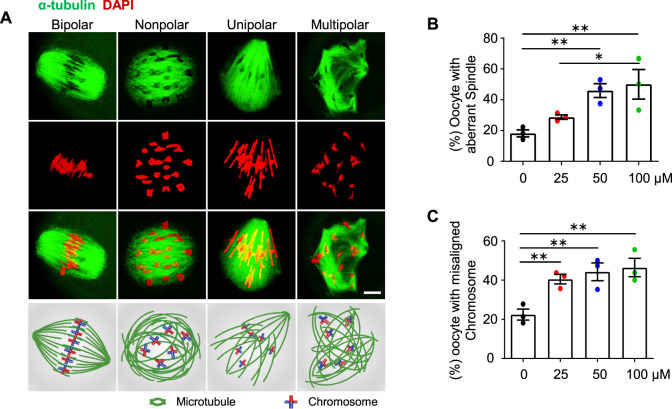
AAI exposure causes aberrant spindle organization and misaligned chromosomes in mouse oocytes. **(A)** GV oocytes were matured in the M16 medium with 0, 25, 50, and 100 μM AAI for 8 h to reach MI. Representative images showing spindle morphology (green) and chromosome alignment (red) in MI oocytes and corresponding illustration at the bottom row. Normally, homologous chromosomes align on the equatorial plate and spindles from opposite poles (“bipolar” spindles, leftmost) attach to the kinetochore of homologous chromosomes at MI. However, aberrant spindle morphologies (“nonpolar”, “unipolar”, and “multipolar” spindles) were also observed. Scale bar, 10 μm. **(B,C)** Quantification of oocytes with aberrant spindles **(B)** and misaligned chromosomes **(C)** in **(A)**. Error bar, mean ± SEM of 3 independent experiments. ∼20 MI oocytes per treatment in each experiment. Totally, 43, 49, 50, and 40 MI oocytes were examined in 0, 25, 50, and 100 μM AAI treatments, respectively. Only comparisons with significant differences were indicated; *, *p* < 0.05; **, *p* < 0.01; one-way ANOVA and the LSD test **(B,C)**.

Data were presented as mean ± SEM. The statistical significance of the differences was determined by the two-tailed *t*-test (Figures 1C,E,G, 2C, and Supplementary Figures S1, S3), one-way ANOVA and followed by Least Significance Difference (LSD) test (Figures 2B, 3B,D, 4B,C, 6A,C,D,E,G,H, 8B,D,F,H, 9A,C,E, and Supplementary Figure S2) or Chi-square (Figure 3E) using SPSS software (SPSS version 20.0; IBM Corporation, NY). Only comparisons with significant differences were indicated: *, *p* < 0.05; **, *p* < 0.01; ***, *p* < 0.001.

## Results

### AAI exposure to oocytes decreases the fertilization rate and embryonic development potential in mice

To investigate the effects of AAI exposure on oocyte quality, an *in vitro* culture system of mouse oocytes was used (Figure 1A). In this system, the fully grown GV oocytes were isolated from mice subjected to superovulation and matured to the metaphase II (MII) stage in the M16 medium in the absence or presence of AAI. The MII oocytes were then *in vitro* fertilized by capacitated sperm, and cultured to the stage of two pronuclei in an HTF medium. The zygotes then develop into two-cell embryos and blastocysts in the KSOM medium. Under the standard culture condition, the oocytes develop to a specific stage at a certain time point (Li S. et al., 2012). Consequently, the samples were collected at appropriate time points to evaluate the oocyte quality (Figure 1A).

The fertilization rate and blastocyst formation rate are two critical assessment criteria for determining the quality of mature oocytes (Rosen et al., 2010; Rienzi et al., 2012; Keefe et al., 2015). Fertilization can be evaluated by the appearance of the pronucleus (Bianchi and Wright, 2016). We took advantage of IVF, in combination with early embryo culture, to examine the rate of two pronuclei and their capability of developing into two-cell embryos and blastocysts. The fully grown GV oocytes were cultured *in vitro* for 12 h. MII oocytes with PB1 were selected and incubated with capacitated sperm. After 6 h of incubation, the rate of two pronuclei reached 77.14% in the control group (0 μM AAI). However, the rate significantly decreased to 61.90% in the 50 μM AAI treatment group (Figure 1B,C). To further confirm this result, these samples were transferred to KSOM embryonic culture medium. After 18 h, the rates of two-cell embryos were examined and found to be 73.01 and 59.52% in the control and 50 μM AAI treatment groups, respectively (Figure 1D,E). The rates of two-cell embryos were closely matched with the rates of two pronuclei, separately, in both the control and AAI treatment groups (73.01 vs. 77.14%; 59.52 vs. 61.90%) (Figure 1C,E). Overall, the fertilization capability of the MII oocytes that matured *in vitro* in the medium containing AAI was significantly decreased. Notably, most of the fertilized MII oocytes (oocytes with two pronuclei) successfully developed into two-cell embryos in both the control and AAI treatment groups. After further examination, we found that the blastocyst formation rate was significantly decreased in the 50 μM AAI treatment group when compared with the control group (20.48 vs. 55.74%) (Figure 1F,G). This suggests that the embryo’s development potential was impaired in the oocytes exposed to AAI.

### AAI exposure disrupts the first polar body (PB1) extrusion in mouse oocytes

The decreased fertilization rate and embryonic development potential suggest poor oocyte quality. The first polar body extrusion (PBE) is also an important marker of oocyte development (Wang and Sun, 2007). In the control group, 83.89% of GV oocytes extruded PB1 after 12 h of *in vitro* maturation (Figure 2A,B). However, the PBE rate was significantly reduced to 66.67% in the 25 μM AAI treatment group. When the concentrations of AAI increased to 50 μM and 100 μΜ, the rates of PBE further decreased (albeit slightly) to 64.13 and 58.61%, respectively (Figure 2A,B). Subsequently, we wondered whether AAI can affect the resumption of meiosis, which is indicated by GVBD (Eppig, 1982). In the control group, nearly all (96%) oocytes completed GVBD at 3 h when cultured *in vitro*. When exposed to 25 μM, 50 μM, or 100 μM AAI, the rates of GVBD reached 92%, which was a slight but not significant decrease when compared with the rates of the control (Figure 2C). These results indicate that AAI exposure to GV oocytes seems not significantly affect the resumption of meiosis, but significantly decreases the rate of PBE.

### AAI exposure induces a high frequency of aneuploidy in mouse oocytes

Given the decreased rate of PBE, we wondered whether AAI exposure would increase the frequency of aneuploidy in oocytes, which is the leading cause of declining oocyte quality in humans (Nagaoka et al., 2012). We then performed the chromosome spread for MII oocytes that matured *in vitro* from GV oocytes, and counted the chromosome number after DAPI staining (Figure 3A). In the control group, most of the MII oocytes showed 20 chromosomes, and the frequency of aneuploidy, which is an oocyte with less than or more than 20 chromosomes, was 21.68% (Figure 3A,B). However, the frequency of aneuploidy was significantly increased to 41.55% in the 25 μM AAI treatment group (Figure 3B). The frequencies of aneuploidy were further increased to 56.02 and 65.95% in the 50 and 100 μM AAI treatment groups, respectively (Figure 3B). This suggests that AAI exposure significantly increases the frequency of aneuploidy in oocytes in a dosage-dependent manner (Figure 3B). These aneuploidies seen at MII are most likely due to the segregation errors of homologous chromosomes in meiosis I. Therefore, our results suggest that AAI exposure dose-dependently induces homologous chromosome mis-segregation in oocytes during meiosis I.

Proper homologous chromosome segregation at meiosis I requires crossovers (COs)/chiasmata, which establish the physical connections between homologous chromosomes (Hunter, 2015; Zickler and Kleckner, 2015). The number and position of COs/chiasmata are strictly controlled, and chromosomes with vulnerable CO/chiasma configurations tend to be mis-segregated (Cole et al., 2012; Nagaoka et al., 2012; Wang et al., 2017). In light of this, the number and position of chiasmata were examined in MI oocytes. The oocytes exposed to AAI showed a significantly decreased number of chiasmata compared with that of the control (Figure 3C,D). Moreover, the higher concentration of AAI, the larger decrease in the number of chiasmata (Figure 3D). Consistently, with the increase in AAI concentration, the proportion of chromosomes with two chiasmata significantly decreased (0 μM: 42.29%; 25 μM: 36.19%; 50 μM: 32.65%; 100 μM: 27.89%), accompanied by a corresponding increase in the proportion of chromosomes with a single chiasma (0 μM: 57.71%; 25 μM: 63.81%; 50 μM: 67.35%; 100 μM: 72.11%) (Figure 3E). Interestingly, it seemed that this alteration largely resulted from the increased proportion of chromosomes with a single terminal but not interstitial chiasma in AAI-exposed oocytes (Figure 3E). These results support the idea that AAI exposure affects the number and position of chiasmata in oocytes.

### AAI exposure causes aberrant spindle organization and chromosome misalignment in mouse oocytes

Proper spindle organization is essential for accurate homologous chromosome segregation (Bennabi et al., 2016). We, therefore, further examined the spindle organization in MI oocytes by immunostaining α-tubulin with a specific antibody (e.g., Zhu et al., 2018; Han et al., 2020). In the control group, most of the MI oocytes showed bipolar spindles (Figures 4A,B, leftmost). However, after AAI exposure, there were significantly fewer proportions of MI oocytes with bipolar spindles, and correspondingly, there were significantly more proportions of oocytes with aberrantly organized spindles including nonpolar, unipolar, and multipolar spindles (Figures 4A,B). Consistent with the dosage-dependent effect of AAI on chromosome mis-segregation (aneuploidy) (Figures 3A,B), the proportion of oocytes bearing aberrant spindles significantly increased with increasing concentrations of AAI exposure (0 μM: 19.44%; 25 μM: 31.83%; 50 μM: 45.94%; 100 μM: 50.74%, Figure 4B). Moreover, a dosage-dependent effect of AAI exposure on chromosome misalignment was also observed (0 μM: 22.62%; 25 μM: 42.16%; 50 μM: 44.22%; 100 μM: 48.06%, Figure 4C).

**FIGURE 5:**
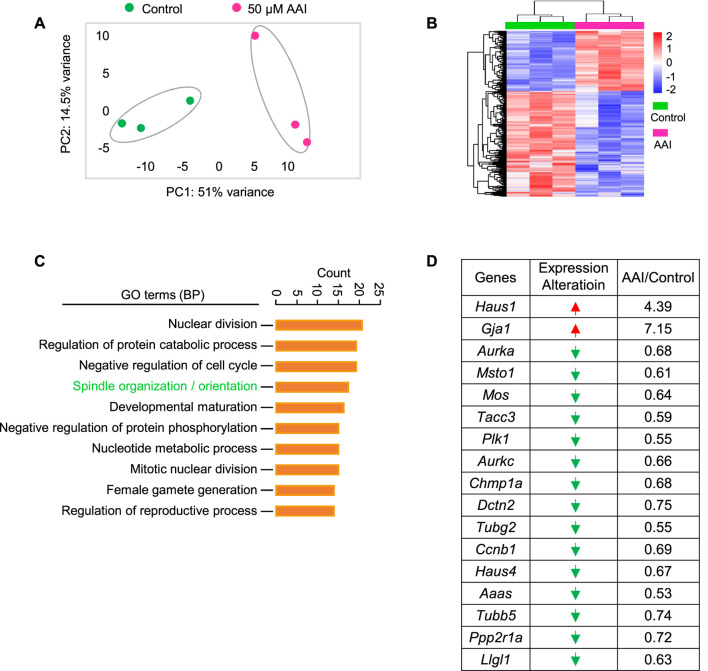
AAI exposure disturbs the expression of genes involved in spindle organization/orientation in GVBD oocytes. **(A)** The fully grown GV oocytes were matured in the M16 medium containing 0 or 50 μM AAI for 2 h to reach the GVBD stage. Oocytes were collected for RNA-seq. PCA analysis of RNA-seq data grouped controls and AAI treatments into two separate clusters. Each data point represents one experiment. **(B)** The heatmap of differentially expressed genes (DEGs) between the control and AAI-exposed groups. **(C)** The top 10 GO-BP terms of DEGs. **(D)** The list of DEGs in the spindle assembly/orientation term in **(C)**. Red arrow, upregulated; green arrow, downregulated.

To further understand how AAI exposure impairs spindle organization in MI oocytes, RNA-seq was performed using GVBD oocytes that matured *in vitro* in the presence or absence of AAI (Figure 5A). In both control and AAI treatment groups, the average correlation coefficient of three repeats in each group was 0.99. In total, 20,362 and 20,620 genes were detected in the control and 50 μM AAI treatment groups, respectively. Among them, 250 genes were upregulated and 436 genes were downregulated after AAI exposure (Figure 5B and Supplementary Table S2). We validated the sequencing results using RT-qPCR (Supplementary Figure S1), and both analyses yielded consistent results. Hence, the reliability of RNA-seq was deemed satisfactory. For GO enrichment analysis, 39 pathways were enriched, and we displayed the top 10 (Figure 5C). Notably, spindle organization/orientation (involving 17 DEGs) was in the top 4 pathways that were significantly altered in the AAI treatment group (Figure 5C, Supplementary Figure S1A, and Supplementary Table S2), and the 17 genes involved are listed (Figure 5D). These results further support the observed defects in spindle morphology and chromosome misalignment in MI oocytes, which would contribute to the observed aneuploidy in MII oocytes following AAI exposure.

### AAI exposure decreases mitochondrial membrane potential (MMP) and ATP production

**FIGURE 6:**
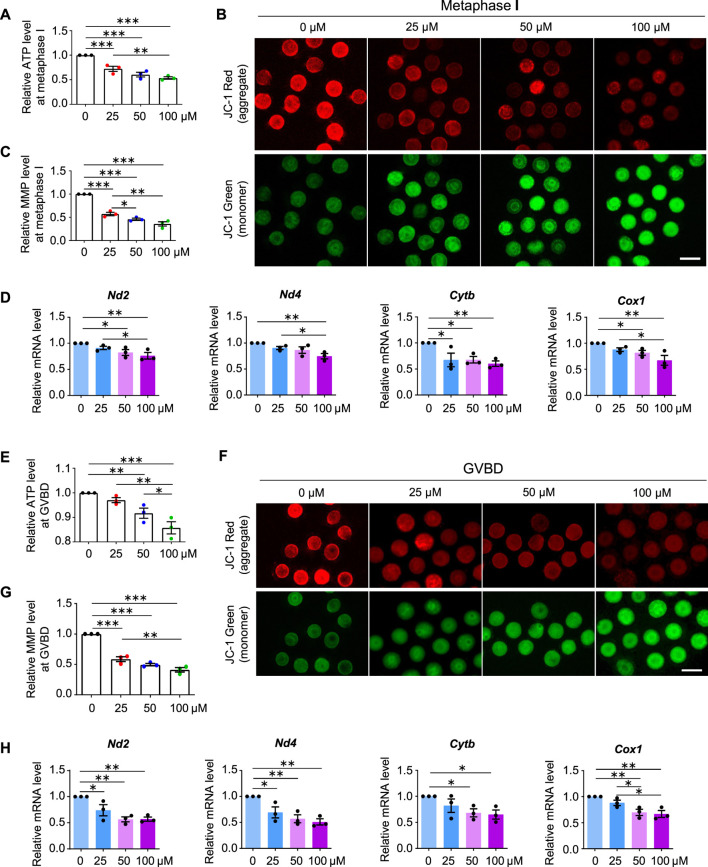
AAI exposure decreases mitochondrial membrane potential (MMP) and ATP production. **(A)** The GV oocytes were matured for 8 h to reach MI in the M16 medium with 0, 25, 50, and 100 μM AAI. Oocytes were collected and the firefly luciferase assay was performed to examine the ATP content. The relative ATP contents in MI oocytes matured in different treatments were shown. **(B,C)** Representative images of JC-1 assay **(B)** and quantification **(C)** of the MMP in MI oocytes. MMP was calculated as the intensity ratio of red fluorescence (J-aggregate) to green fluorescence (J-monomer). **(D)** Relative expression levels of selected genes associated with mitochondrial respiratory chain in MI oocytes by RT-qPCR. **(E–H)** The relative ATP content, MMP, and relative mRNA levels of mitochondrial respiratory chain-related genes were examined in GVBD oocytes as in MI oocytes **(A–D)**. Scale bar, 100 μm **(B,F)**. Error bar, mean ± SEM of 3 independent experiments **(A,C,D,E,G,H)**. 10 oocytes were used for ATP assay for each experiment per treatment and totally 30 oocytes were used for each treatment **(A,E)**. ∼15 oocytes were used for JC-1 assay for each experiment per treatment and totally 43, 48, 47, and 46 oocytes in **(C)** and 50, 48, 50, and 39 oocytes in **(G)** were used for each treatment. 25 oocytes were used for each experiment per treatment, and totally 75 oocytes were used for each treatment **(D,H)**. Only comparisons with significant differences were indicated; *, *p* < 0.05; **, *p* < 0.01; ***, *p* < 0.001; one-way ANOVA and the LSD test.

Normal spindle assembly requires sufficient ATP supply (Zhang et al., 2006; Dalton and Carroll, 2013). Therefore, we considered whether aberrant spindles also resulted from reduced ATP production in AAI-exposed oocytes. To test this idea, we examined the ATP content in MI oocytes using the classical firefly luciferase. In the presence of ATP and Mg^2+^, firefly luciferase catalyzes luciferin oxidation to yield oxyluciferin, which emits luminescence (Inouye, 2010). Compared with the control, AAI exposure significantly reduced the ATP content in MI oocytes (Figure 6A).

MMP is required to maintain mitochondrial oxidative phosphorylation to produce ATP (Zorova et al., 2018). The lipophilic cationic fluorescent dye JC-1 has been widely used to measure MMP, which is positively correlated with the level of JC-1 aggregates and thus can be measured by the ratio of red (from JC-1 aggregates) to green (from JC-1 monomers) fluorescence (Sivandzade et al., 2019). A significant decrease in the intensity of red/green JC-1 fluorescence was observed in MI oocytes that matured *in vitro* in the medium containing AAI (Figures 6B,C).

Impaired MMP and reduced ATP production further suggest that the mitochondrial respiratory chain may be disturbed. Mitochondrial genome-encoded NADH dehydrogenase subunit 2 (ND2), NADH dehydrogenase subunit 4 (ND4), Cytochrome B (CYTB), and Cyclooxygenase 1 (COX1) are essential components of the mitochondrial respiratory chain (Poyton and McEwen 1996). Our RT-qPCR results showed that AAI exposure decreased the mRNA levels of these genes in MI oocytes (Figure 6D).

Further investigation showed that decreased MMP and ATP levels were observed in oocytes at the GVBD stage after AAI exposure (Figures 6E–G). Moreover, reduced expression of mitochondrial genome-encoded ND2, ND4, CYTB, and COX1 were also seen in oocytes at this stage (Figure 6H). Notably, significantly decreased expression of gene encoding CYTB was observed in GV oocytes exposed to AAI for 1 h (Supplementary Figure S2). CYTB is essential for the assembly and function of complex III of the mitochondrial respiratory chain, and it forms the catalytic core of the enzyme together with the other two components (Blakely et al., 2005). CYTB disruption can cause severe mitochondrial respiratory chain enzyme deficiency in humans and yeast (Blakely et al., 2005). These results support that the mitochondria of oocytes became impaired after AAI exposure.

**FIGURE 7:**
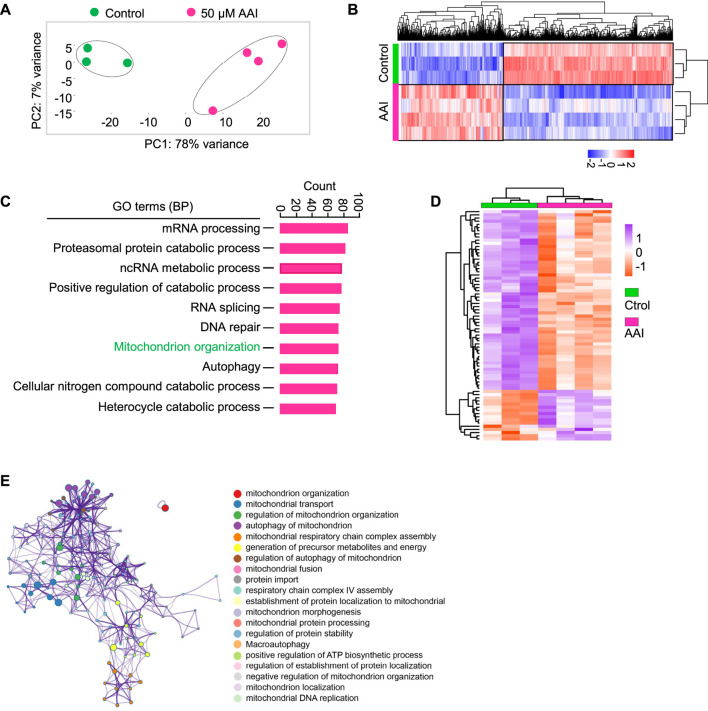
AAI exposure alters the expression levels of genes involved in mitochondrion organization. **(A)** The GV oocytes were matured in the M16 medium containing 0 or 50 μM AAI for 8 h to reach MI stage. The oocytes were collected for RNA-Seq. PCA analysis of RNA-seq data grouped controls and AAI treatments into two separate clusters. Each data point represents one experiment. The correlation coefficient is 0.99 and 0.97 between three repeats in control and four repeats in AAI treatment, respectively. **(B)** The heatmap of DEGs between the control and AAI-exposed groups. **(C)** The top 10 GO-BP terms of DEGs. **(D)** The heatmap of DEGs enriched in mitochondrion organization in **(C)**. **(E)** The annotation of DEGs for mitochondrion organization in **(D)**.

Interestingly, GO and KEGG pathway analyses did not enrich the nuclear genome encoding the mitochondria-related pathway at the GVBD stage after AAI exposure (Supplementary Table S2). To further explore the effect of AAI exposure, we performed RNA-seq using MI oocytes that matured *in vitro* in the presence or absence of AAI (Figure 7A). Among them, 990 genes were upregulated and 1,585 genes were downregulated after AAI exposure (Figures 7A,B, Supplementary Table S3). We validated the RNA-seq results using RT-qPCR (Supplementary Figure S3), and both analyses yielded consistent results. Hence, the reliability of the RNA-seq of MI oocytes was deemed satisfactory. For the GO pathway analysis, 40 pathways were significantly altered in the AAI treatment group. Notably, mitochondrion organization (involving 73 DEGs) was among the top 7 significantly altered pathways (Figure 7C). Of the 73 DEGs, 16 were upregulated and 57 were downregulated in the AAI treatment group compared with the control (Figure 7D). These DEGs were involved in 20 processes related to the mitochondria (Figure 7E). Overall, these results support the idea that AAI exposure impairs mitochondrial function in oocytes.

### AAI exposure increases ROS levels and induces early apoptosis in mouse oocytes

**FIGURE 8:**
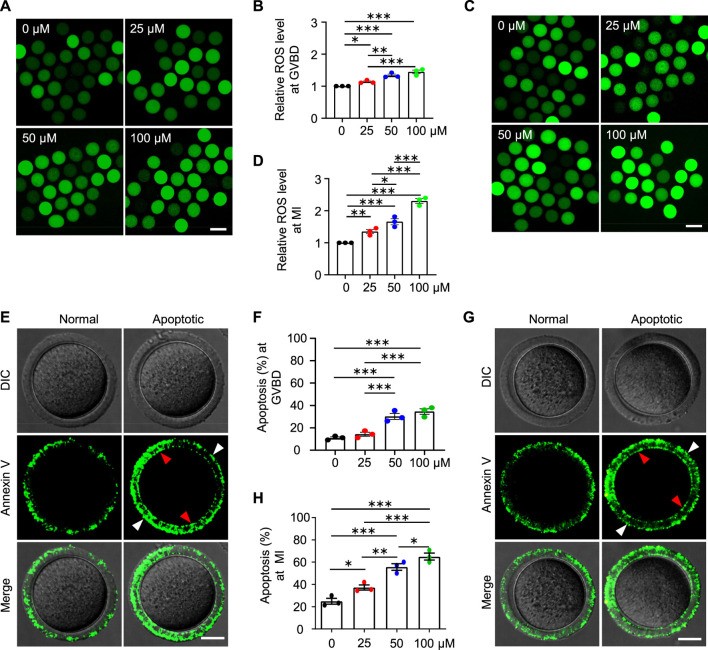
AAI exposure increases intracellular ROS levels and induces early apoptosis in mouse GVBD and MI oocytes. **(A–D)** The GV oocytes were matured for 2 and 8 h to reach GVBD and MI stage in the M16 medium with 0, 25, 50, and 100 μM AAI. Oocytes were collected and ROS levels were examined with the reliable DCFH-DA probe. Representative images showing ROS levels in GVBD oocytes **(A)**. Quantification of **(A)** showing the relative ROS level **(B)**. Representative images showing ROS levels in MI oocytes **(C)**. Quantification of **(C)** showing the relative ROS level **(D)**. Scale bar, 100 μm **(A,C)**. Error bar, mean ± SEM of 3 independent experiments. ∼30 oocytes per treatment in each experiment. Totally 96, 74, 86, and 87 GVBD oocytes and 85, 76, 90, and 71 MI oocytes were used for each treatment, respectively **(B,D)**. *, *p* < 0.05; **, *p* < 0.01; ***, *p* < 0.001; one-way ANOVA and the LSD test **(B,D)**. **(E,G)** Representative images showing Annexin V staining of GVBD oocytes **(E)** or MI oocytes **(G)** that matured in the M16 medium with indicated AAI concentration. The detection of early apoptosis was performed using FITC conjugated Annexin V. Oocytes with positive Annexin V staining on both zona pellucida (white arrowheads) and cytoplasmic membrane (red arrowheads) were considered early apoptosis. Scale bar, 20 μm **(E,G)**. **(F,H)** Quantification of **(E,G)** showing the rates of early apoptotic oocytes. Error bar, mean ± SEM of 3 independent experiments. 25–30 oocytes per treatment in each experiment. Totally, 73, 85, 85, 81 GVBD oocytes and 88, 91, 70, and 77 MI oocytes were used for each treatment, respectively **(F,H)**. Only comparisons with significant differences were indicated; *, *p* < 0.05; **, *p* < 0.01; ***, *p* < 0.001; one-way ANOVA and the LSD test **(F,H)**.

Mitochondria also produce low levels of reactive oxygen species (ROS), which are involved in many processes including signaling transduction and immune response (e.g., Hamanaka and Chandel 2010). However, impaired mitochondria may produce excessive ROS. We, therefore, examined the ROS level in the oocytes at different stages with the commonly used and reliable probe DCFH-DA (2′,7′-Dichlorodihydrofluorescein diacetate) (Figures 8A,C). DCFH-DA diffuses into cells and is deacetylated by esterases to form DCFH (2′,7′-dichlorodihydrofluorescein), which is then oxidized to 2′,7′-dichlorofluorescein (DCF) by ROS (predominantly H_2_O_2_) (McLennan and Degli Esposti, 2000). DCF emits fluorescence, which can be detected and quantified. As expected, the ROS levels in the AAI-exposed GVBD and MI oocytes were significantly increased compared with those of the control (Figures 8A–D). Since both excessive ROS and mitochondrial dysfunction can induce apoptosis (Simon et al., 2000), we further evaluated early apoptosis in GVBD and MI oocytes *via* the Annexin V staining assay (Figures 8E,G). During early apoptosis, phosphatidylserine, normally located on the inner surface of the membrane, is exposed on the outside surface and bound to anticoagulant protein Annexin V with high affinity (van Engeland et al., 1998). The proportion of GVBD oocytes with positive Annexin V staining was 10.90% in the control group (Figures 8E,F). However, the proportions of Annexin V positive GVBD oocytes were significantly increased to 14.26, 30.20, and 34.52% for oocytes treated with 25 μM, 50 μM, and 100 μM AAI, respectively (Figures 8E,F). Notably, the proportion of MI oocytes with positive Annexin V staining was 24.92% in the control group (Figures 8G,H). However, the proportions of Annexin V positive MI oocytes were significantly increased to 37.30, 55.71, and 65.04% for oocytes treated with 25 μM, 50 μM, and 100 μM AAI, respectively (Figures 8G,H). This result indicates that AAI exposure can dosage-dependently induce early apoptosis in GVBD and MI oocytes.

**FIGURE 9:**
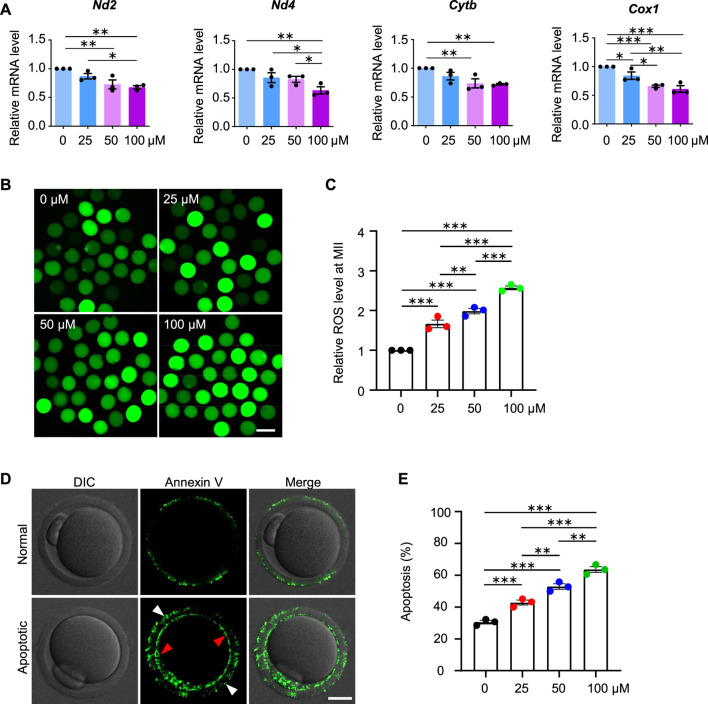
AAI exposure increases intracellular ROS levels and induces early apoptosis in mouse MII oocytes. **(A–E)** The GV oocytes were matured for 12 h to reach MII in the M16 medium with 0, 25, 50, and 100 μM AAI. Relative expression levels of selected genes associated with mitochondrial respiratory chain in MII oocytes by RT-qPCR **(A)**. ROS levels in MII oocytes were examined with the reliable DCFH-DA probe **(B)**. Quantification of **(B)** showing the relative ROS level **(C)**. Representative images showing Annexin V staining of MII oocytes from different treatments **(D)**. Quantification of **(D)** showing the percentage of apoptosis **(E)**. Scale bar, 100 μm in **(B)**; 20 μm in **(D)**. Error bar, mean ± SEM of 3 independent experiments. 25 oocytes were used for each experiment per treatment, and totally 75 oocytes were used for each treatment **(A)**. ∼25 oocytes per treatment in each experiment and totally 73, 76, 79, and 85 MII oocytes were used for each treatment, respectively **(C)**. 30–35 oocytes per treatment in each experiment and totally 92, 91, 100, and 102 oocytes were used for each treatment **(E)**. Only comparisons with significant differences were indicated; *, *p* < 0.05; **, *p* < 0.01; ***, *p* < 0.001; one-way ANOVA and the LSD test **(C,E)**.

Given the decreased fertilization rate and embryo developmental potential, we further examined the oocytes at the MII stage and found decreased mRNA expression for genes involved in the mitochondrial respiratory chain, increased ROS levels and early apoptosis in AAI treatment groups (Figure 9), which is consistent with the results from the oocytes at the GVBD and MI stages. These results further confirm the poor quality of oocytes after AAI exposure.

## Discussion

**FIGURE 10:**
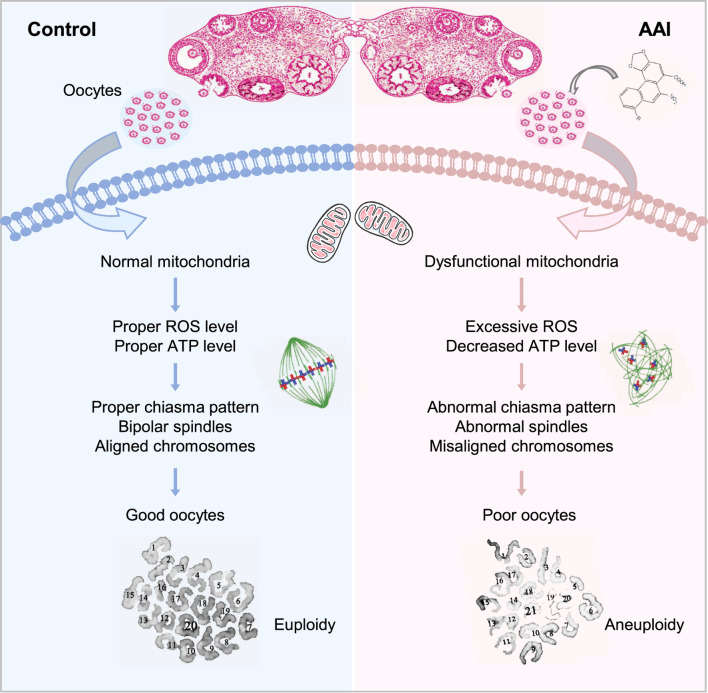
Model for the impact of AAI exposure on oocyte quality. AAI exposure impairs mitochondrial function and metabolism, leading to insufficient ATP supply. As a result, spindle organization and chromosome arrangement in MI oocytes are disturbed, which leads to chromosome segregation errors and ultimately oocytes with poor quality (e.g., aneuploidy).

Our investigation reveals that AAI exposure dramatically increases the frequency of aneuploidy in oocytes, and significantly decreases the rate of PBE, fertilization capability, and embryo development potential. Further analysis suggests that these defects are most likely caused by mitochondrial dysfunction, as revealed by the insufficient ATP supply and excessive ROS (Figure 10).

### AAI impairs oocyte maturation

In contrast with male meiosis, which is a continuous process, female meiosis begins from fetal development and is arrested at diplotene/dictyate soon after CO formation. Arrested oocytes grow and are gradually surrounded by granulosa cells to form follicles. From puberty, under the role of LH, a small number of oocytes resume meiosis, and gradually progress to MII when they are ready to be fertilized (Morelli and Cohen, 2005; Bolcun-Filas and Handel, 2018). During this process of oocyte maturation, oocytes are exposed to the stimulation of both internal and external factors (Li et al., 2019; Li et al., 2020).

Although AAI exposure to mouse GV oocytes only slightly decreases the rate of GVBD, the RNA-seq of GVBD oocytes shows that AAI exposure significantly affects many biological processes related to oocyte maturation. Further study shows that AAI exposure significantly decreases the rate of PB1 extrusion, indicating that a fraction of oocytes probably fail to complete meiosis I. Even if the majority of oocytes can progress to the MII stage, the proportion of MII oocytes that successfully formed pronuclei was significantly decreased when they were *in vitro* fertilized, suggesting that AAI exposure also impairs the fertilization capability of MII oocytes. Interestingly, most MII oocytes with pronuclei could successfully develop into two-cell embryos as the proportions of MII oocytes with pronuclei and two-cell embryos were essentially equal in both control and AAI treatment groups. However, the rate of blastocyst formation was dramatically decreased in the AAI-exposed oocytes when compared with the oocytes in the control group. Overall, these results suggest that AAI exposure impairs the oocyte maturation process.

Exposure of mouse oocytes to AAI results in mitochondrial dysfunction, which includes decreased MMP, insufficient ATP supply, and excessive ROS. Elevated ROS levels have also been observed in AAI-exposed porcine oocytes and human mitotic cells (Yu et al., 2011; Romanov et al., 2015; Zhang Y. et al., 2019). ATP deficiency and excessive ROS could disturb the assembly of spindle microtubules, as well as other intracellular organelles and the cytoskeleton (Dumollard et al., 2003; Eichenlaub-Ritter et al., 2004). Furthermore, the disturbance of spindle assembly can cause chromosome misalignment, ultimately resulting in aneuploidy, as observed. Impaired mitochondria and defective oocyte maturation can decrease fertilization capacity and embryo developmental potential (Reynier et al., 2001; Cummins, 2002). Therefore, defects in oocyte maturation induced by AAI exposure seem to largely result from mitochondrial dysfunction (Figure 10). However, we cannot exclude the possibility that AAI may impair different events in different ways.

The above results raise interesting questions: how AAI enters oocytes and how AAI affects mitochondria. It has been proposed that AA is absorbed from the gastrointestinal tract, enters the bloodstream, and is distributed throughout the body since DNA adducts have been found in various organs, including the kidney, bladder, liver, stomach, intestine, and lung (Mei et al., 2006; Shibutani et al., 2007; Dickman et al., 2011; Jadot et al., 2017). Studies on AA-mediated nephrotoxicity suggest that proteins of the organic anion transporter (OAT) family (OAT1, OAT2, and OAT3) play an important role in mediating the AA entering proximal tubular epithelial cells in the kidney (Jadot et al., 2017). However, it seems that OAT1, OAT2, and OAT3 are not expressed in mouse ovaries (https://www.ncbi.nlm.nih.gov/gene/). Additionally, our RNA-seq of GVBD and MI oocytes did not detect the expression of these genes. Therefore, AAI may enter oocytes through diffusion or other mediators. Consistent with this idea, AAI has also been observed to be accumulated in CHO-K1 cells, which seem to not express OATs (Dickman et al., 2011).

It has been widely accepted that intracellular AA metabolic intermediates, mainly catalyzed by NAD(P)H: quinone oxidoreductase (NQO1) and/or cytochrome P450 (CYP), covalently bind to DNA and RNA to form adducts (Leung and Chan, 2015; Jadot et al., 2017). The nitroreduction process consumes NADPH and related enzymes that play important roles in scavenging superoxide anion radicals (Zangar et al., 2004; Omura, 2006; Zhu and Li, 2012; Bhattacharyya et al., 2014). Therefore, pathological activation of the reduction process may disrupt the redox balance and cause oxidative stress. It has also been reported that AAI intermediates can react with aminothiols, such as glutathione (GSH), which is an important antioxidant that protects cells from oxidative stress (Zhang et al., 2020). Consistently, AAI exposure leads to GSH depletion in human mitotic cells, which can further cause oxidative stress (Yu et al., 2011; Li Y. C. et al., 2012; Romanov et al., 2015; Zhang et al., 2020). Therefore, AAI exposure may cause oxidative stress in oocytes *via* the same pathways. Furthermore, increased oxidative stress can damage mitochondria and lead to further increases in oxidative stress levels.

### How does AAI exposure affect the chiasma pattern?

Chromosomes with aberrant CO/chiasma configuration are at high risk for chromosome mis-segregation to generate aneuploidy, which is the major cause of infertility, spontaneous abortion, and birth defects (Nagaoka et al., 2012; Wang et al., 2017). Meiotic CO recombination is completed at pachytene before oocytes are arrested (at diplotene/dictyate; Hunt and Hassold, 2008; Hunter, 2015). GV oocytes exposed to AAI did not affect either the numbers or the positions of their COs. However, surprisingly, a dosage-dependent decrease in chiasma number was observed in MI oocytes that matured in the presence of AAI. Moreover, the decrease in chiasma number in AAI-exposed oocytes was largely due to the decreased proportion of chromosomes with two chiasmata, and the increased proportion of chromosomes with a single terminal (but not interstitial) chiasma. Since the maintenance of chiasmata also requires sister cohesion, alterations in chiasma pattern in AAI-exposed oocytes most likely resulted from the partial loss of sister cohesion as is often observed in aged females (e.g., Hodges et al., 2005).

In mice, each chromosome usually has one or two COs/chiasmata. For chromosomes with only one CO/chiasma, the CO/chiasma tends to be located around the middle of the chromosomes (Supplementary Figure S4i) (Wang et al., 2017; Wang et al., 2021). For chromosomes with two COs/chiasmata, usually one is close to the distal end and the other one is close to the proximal end (Supplementary Figure S4ii) (Wang et al., 2017; Wang et al., 2021). When sister cohesion is weakened, the distal chiasma would be preferentially eliminated (but not the CO at the DNA level) since it is held only by minimal cohesion (Supplementary Figure S4iv) (Wang et al., 2017; Wang et al., 2019). AAI exposure increases ROS, which would damage the sister cohesion, as previously reported (Perkins et al., 2016; Perkins et al., 2019). The proximal chiasma would be less affected than the distal chiasma since there is a large number of cohesin around the centromere to hold the sisters together (Supplementary Figure S4iv). As a result, 1) the number of chiasmata per oocyte is decreased, and 2) more chromosomes have a single terminal chiasma. These predictions are consistent with what we observed. In addition to altered chiasma pattern, chromosome mis-segregation would be further exaggerated by other defects, such as the aberrantly arranged spindles observed in AAI-exposed oocytes. The increased frequency of aneuploidy in oocytes would increase the risk of miscarriage and birth defects in offspring, as has been observed in humans (Nagaoka et al., 2012).

## Data Availability

The datasets presented in this study can be found in online repositories. The names of the repository/repositories and accession number(s) can be found below: SRA, PRJNA793336 and PRJNA836407.
