# Supplementary material for: Aristolochic acid I exposure decreases oocyte quality
Source: Front Cell Dev Biol. 2022 Aug 11;10:838992. doi: 10.3389/fcell.2022.838992 (PMC9402977; doi:10.3389/fcell.2022.838992)
Supplement: Supplementary file 1 [file DataSheet4.pdf]

Supplementary Table 1. Primers used for RT-qPCR

Supplementary Table 2. Differential expressed genes in GVBD oocytes and GO-BP/KEGG pathway analysis

Supplementary Table 3. Differential expressed genes in MI oocytes and GO-BP/KEGG pathway analysis

Supplementary Figure 1. AAI exposure disturbs the expression of genes associated with spindle organization in GVBD oocytes.

Supplementary Figure 2. The relative expression levels of genes associated with mitochondrial respiratory chain in GV oocytes by RT-qPCR.

Supplementary Figure 3. AAI exposure disturbs the expression of genes associated with mitochondrial oxidative phosphorylation in MI oocytes.
