# Supplementary material for: Aristolochic acid I exposure decreases oocyte quality
Source: Front Cell Dev Biol. 2022 Aug 11;10:838992. doi: 10.3389/fcell.2022.838992 (PMC9402977; doi:10.3389/fcell.2022.838992)
Supplement: Supplementary file 2 [file Table1.DOCX]

**Supplementary table 1.** Primers used for RT-qPCR

| **Genes** | **Primers** | **Size (bp)** | **Accession number**  **(NCBI)** | **Temperature**  **(°C)** |
| --- | --- | --- | --- | --- |
| *Nd2* | F: CCATTCCACTTCTGATTACC  R: GTCATGTAAGAAGAATAAGTCC | 73 | NC_005089.1 | 60 |
| *Nd4* | F: CCAGCCTAACACTTCTATG  R: GGCTAGCTATTAATATTAGTGGC | 132 | NC_005089.1 | 60 |
| *Cytb* | F: ATTCCTTCATGTCGGACGAG  R: ACTGAGAAGCCCCCTCAAAT | 228 | NC_005089.1 | 60 |
| *Cox1* | F: TTTTCAGGCTTCACCCTAGATGA  R: CCTACGAATATGATGGCGAAGTG | 62 | NC_005089.1 | 60 |
| *Plk1* | F: CTTCGCCAAATGCTTCGAGAT  R: TAGGCTGCGGTGAATTGAGAT | 136 | NM_011121.4 | 60 |
| *Haus1* | F: GTCGCTGAGTGGTTGAAAAAGG  R: GGACCCTGTTGCGTTCTGAA | 109 | NM_146089.3 | 60 |
| *Ccnb1* | F: AAGGTGCCTGTGTGTGAACC  R: GTCAGCCCCATCATCTGCG | 228 | NM_172301.3 | 60 |
| *Aurka* | F: CTGGATGCTGCAAACGGATAG  R: CGAAGGGAACAGTGGTCTTAACA | 105 | NM_011497.4 | 60 |
| *Aurkc* | F: GGGCGTGTGTACTTGGCTC  R: AAGTTGGTGCTCCAATCCCTC | 102 | NM_001080965.1 | 60 |
| *Tubb5* | F: GATCGGTGCTAAGTTCTGGGA  R: AGGGACATACTTGCCACCTGT | 139 | NM_011655.5 | 60 |
| *Ndufa12* | F: ACCGATGGGTCATCTACACCA  R: TCGTCAGTCATGCAGTGAAGC | 112 | NM_025551.4 | 60 |
| *Atp5b* | F: GGTTCATCCTGCCAGAGACTA  R: AATCCCTCATCGAACTGGACG | 120 | NM_016774.3 | 60 |
| *Bcl2l1* | F: GACAAGGAGATGCAGGTATTGG  R: TCCCGTAGAGATCCACAAAAGT | 124 | NM_001289716.1 | 60 |
| *Mapk3* | F: TCCGCCATGAGAATGTTATAGGC  R: GGTGGTGTTGATAAGCAGATTGG | 248 | NM_011952.2 | 60 |
| *Rcc1* | F: ATGCCACCCAAGCGCATAG  R: CAAGCCTGGTTCTGTGTTGTG | 147 | NM_001197082.1 | 60 |
| *Rae1* | F: TTTGGGAGCACAACCACAGAT  R: TAAAGTTGGCGGGCTGAAAGA | 105 | NM_175112.5 | 60 |
| *Fsd1* | F: GAGGGAGGCGCTAAGAAAAAT  R: GAACCGACGTGAGGGACTG | 161 | NM_183178.2 | 60 |
| *Cltc* | F: AGATTCTGCCCATTCGCTTTC  R: TCAGTGCAATCACTTTGCTGG | 240 | [NM_001356393.1](https://www.ncbi.nlm.nih.gov/entrez/viewer.fcgi?db=nucleotide&id=1249618520) | 60 |
| *Psrc1* | F: GCATAAAGAAGGAATCACCCACT  R: CACCGAACCCAGTTTTCCG | 111 | NM_001190161.1 | 60 |
| *Uqcrfs1* | F: GAGCCACCTGTTCTGGATGTG  R: GCACGACGATAGTCAGAGAAGTC | 167 | NM_025710.2 | 60 |
| *Ndufa10* | F: ACCTTTCACTACCTGCGGATG  R: GTACCCAGGGGCATACTTGC | 162 | NM_024197.1 | 60 |
| *Atp6v0b* | F: AGTTGCTCTACCTCGGGATCT  R: ATGCCACATCAAAGCGAAAGC | 102 | NM_033617.3 | 60 |
| *Ndufa1* | F: ATGTGGTTCGAGATTCTCCCT  R: TGGTACTGAACACGAGCAACT | 131 | NM_019443.2 | 60 |
| *Ndufb3* | F: ACAGACAGTGGAAAATTGAAGGG  R: GCCCATGTATCTCCAAGCCT | 110 | NM_025597.3 | 60 |
| *Gapdh* | F: GACAAAATGGTGAAGGTCGGT  R: GAGGTCAATGAAGGGGTCG | 120 | NM_001289726.1 | 60 |
