# Supplementary material for: Aristolochic acid I exposure decreases oocyte quality
Source: Front Cell Dev Biol. 2022 Aug 11;10:838992. doi: 10.3389/fcell.2022.838992 (PMC9402977; doi:10.3389/fcell.2022.838992)
Supplement: Supplementary file 3 [file DataSheet3.pdf]

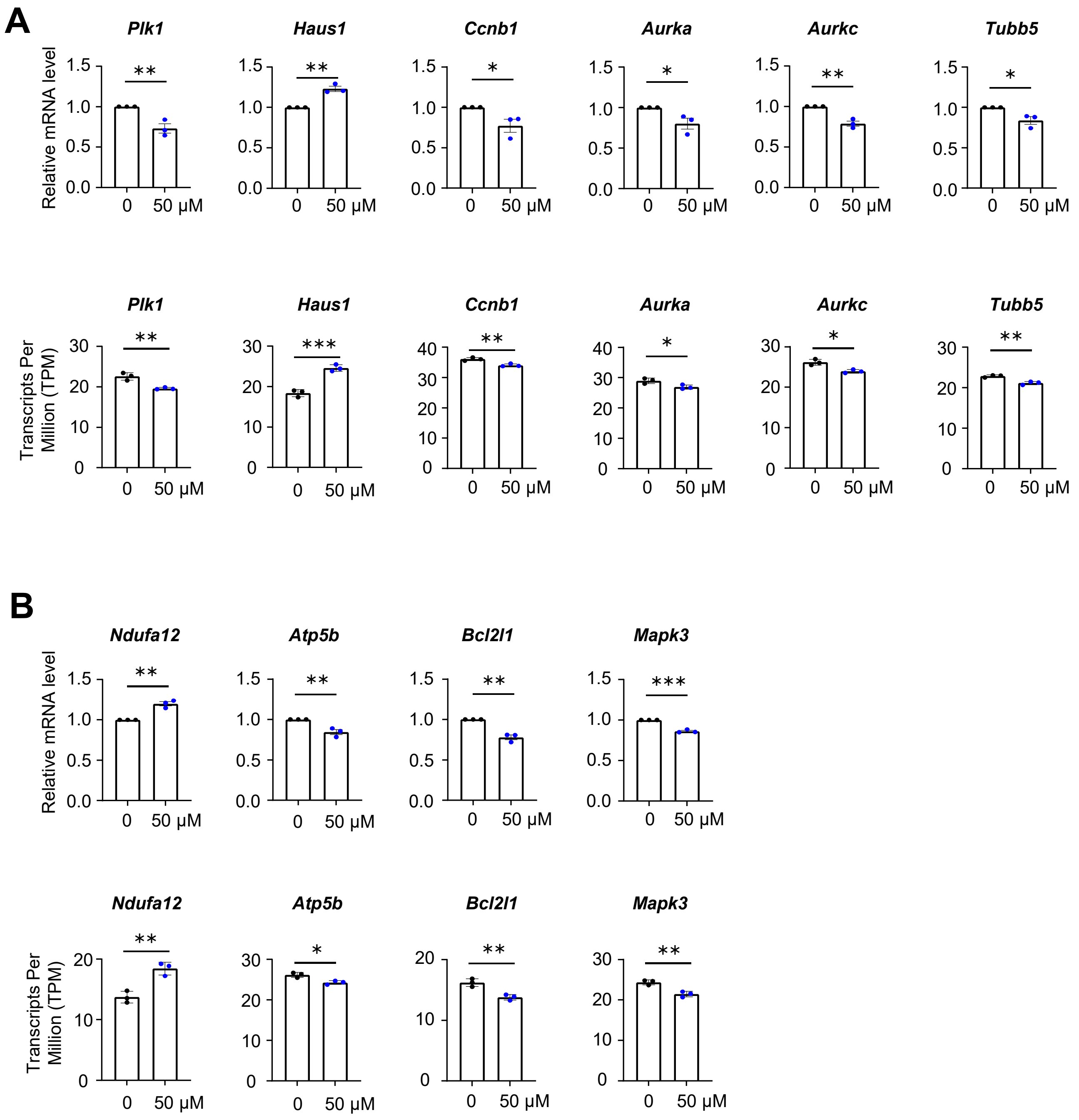

Supplementary Figure 1. AAI exposure disturbs the expression of genes associated with spindle organization in GVBD oocytes.

(A, B) The mRNA levels of genes related with spindle organization (A), oxidative phosphorylation and apoptosis (B) were examined by RT-qPCR (top) and RNA-seq (bottom). Error bar, mean ± SEM of 3 independent experiments. *, $p < 0.05$; **, $p < 0.01$; ***, $p < 0.001$; two-tailed t-test.

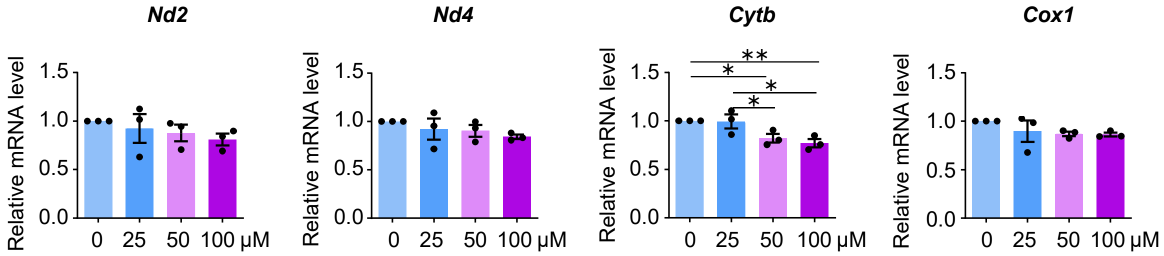

Supplementary Figure 2. The relative expression levels of genes associated with mitochondrial respiratory chain in GV oocytes by RT-qPCR.

The GV oocytes were cultured in M16 medium containing 0, 25, 50 or 100 μM AAI for 1h. The relative expression levels of selected genes in mitochondrial respiratory chain by RT-qPCR. Error bar, mean ± SEM of 3 independent experiments. 25 oocytes were used for each experiment per treatment, and totally 75 oocytes were used for each treatment. Only comparisons with significant differences were indicated; *, $p < 0.05$; **, $p < 0.01$; one-way ANOVA and the LSD test.

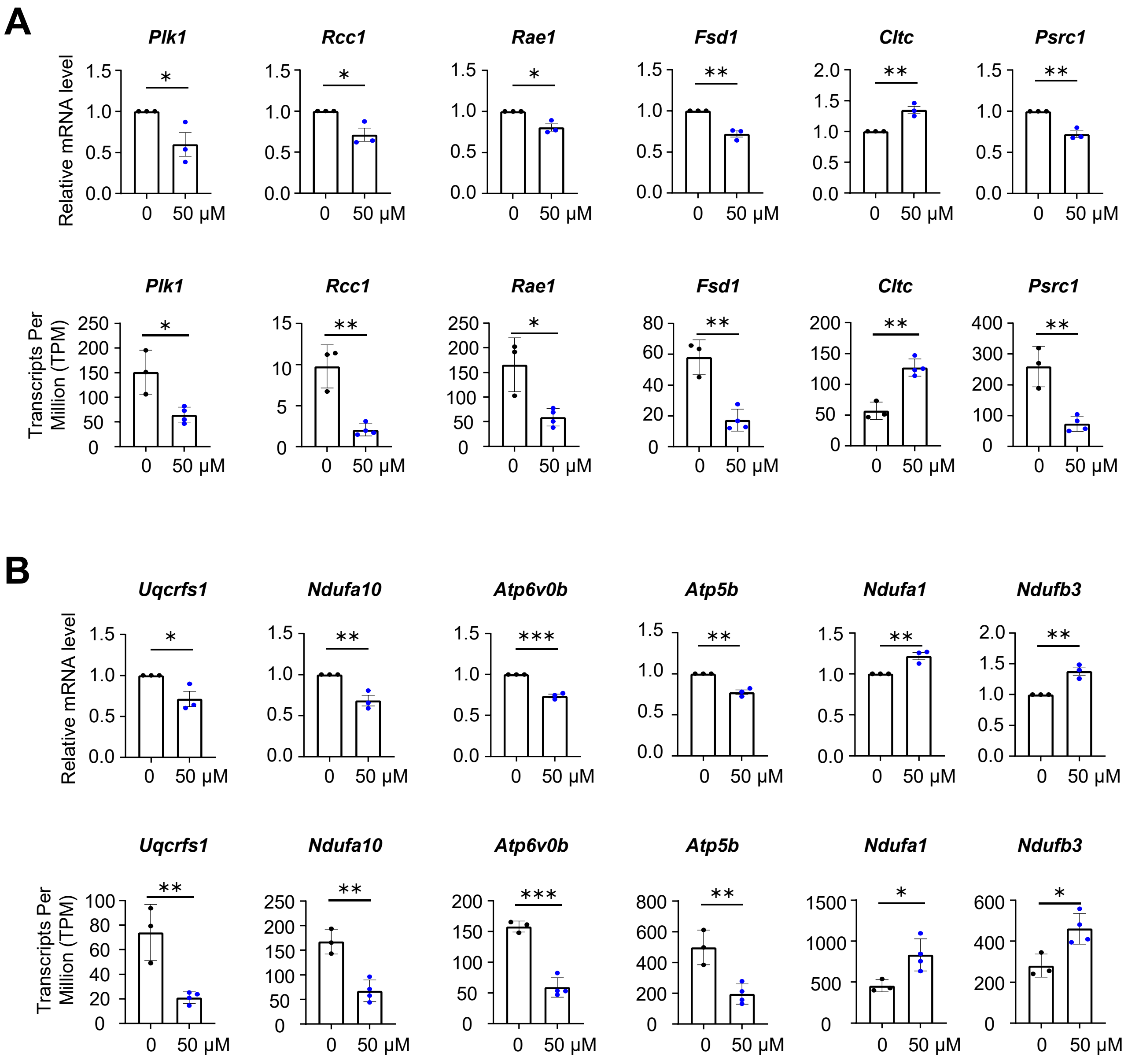

Supplementary Figure 3 AAI exposure disturbs the expression of genes associated with mitochondrial oxidative phosphorylation in MI oocytes.

(A, B) The mRNA levels of genes associated with oxidative phosphorylation (A) and spindle assembly (B) from RT-qPCR (top) or RNA-seq (bottom). Error bar, mean ± SEM of at least 3 independent experiments. *, $p < 0.05$; **, $p < 0.01$; ***, $p < 0.001$; two-tailed t-test.

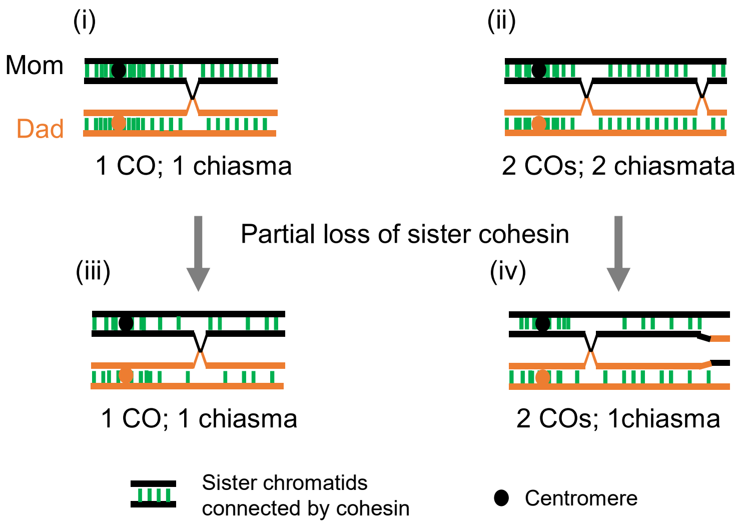

Supplementary Figure 4. Chiasma pattern on the chromosomes.

Each mouse chromosome has one or two COs/chiasmata. For chromosomes with only one CO/chiasma, the CO/chiasma tends to be located around the middle of chromosomes (i). For chromosomes with two COs/chiasmata, usually one is close to the distal end and the other one is close to the proximal end due to CO interference (pushing two or more COs stay far away each other) (ii). Premature loss of sister cohesion, e.g. damaged by excess ROS, preferentially eliminates the distal chiasma since it is maintained only by a minimal cohesin (iv). The proximal chiasma (iii) and interstitial chiasma (iv) are maintained by a large number of cohesin and thus less affected.
